# LSD1/KDM1A inhibitors in clinical trials: advances and prospects

**DOI:** 10.1186/s13045-019-0811-9

**Published:** 2019-12-04

**Authors:** Yuan Fang, Guochao Liao, Bin Yu

**Affiliations:** 1 Joint Laboratory for Translational Cancer Research of Chinese Medicine of the Ministry of Education of the People’s Republic of China, International Institute for Translational Chinese Medicine, Guangzhou University of Chinese Medicine, Guangzhou, 510006 Guangdong China; 2 School of Pharmaceutical Sciences, Zhengzhou University, Zhengzhou, 450001 China; 3 State Key Laboratory of Pharmaceutical Biotechnology, Nanjing University, Nanjing, 210023 China

**Keywords:** Epigenetics, Histone demethylase, LSD1 inhibitors, Cancer therapy

## Abstract

Histone demethylase LSD1 plays key roles during carcinogenesis, targeting LSD1 is becoming an emerging option for the treatment of cancers. Numerous LSD1 inhibitors have been reported to date, some of them such as TCP, ORY-1001, GSK-2879552, IMG-7289, INCB059872, CC-90011, and ORY-2001 currently undergo clinical assessment for cancer therapy, particularly for small lung cancer cells (SCLC) and acute myeloid leukemia (AML). This review is to provide a comprehensive overview of LSD1 inhibitors in clinical trials including molecular mechanistic studies, clinical efficacy, adverse drug reactions, and PD/PK studies and offer prospects in this field.

## Introduction

**Fig. 1:**
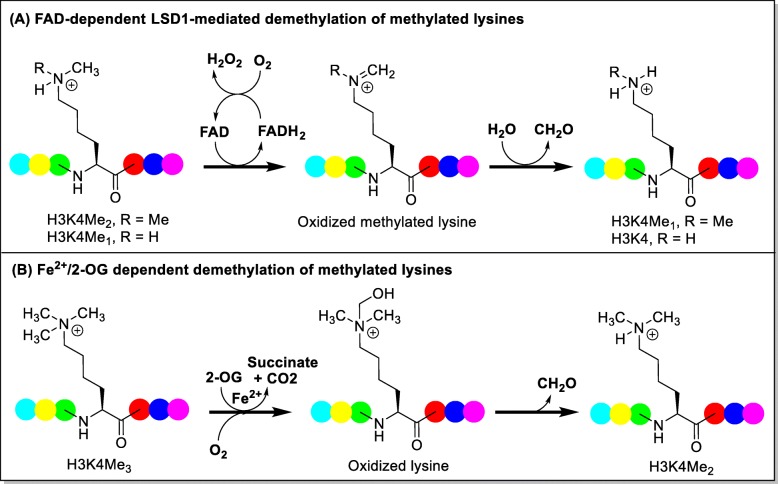
**a**, **b** Histone demethylase enzymes LSD1 and JmjC domain-containing family and their mechanisms of demethylation. Amino acid unit is represented in colored dot

Lysine methyltransferases and demethylases have been reported to be able to catalyze the process of *N*-methylation and *N*-demethylation of histone lysines, respectively [1, 2]. Based on the catalytic mechanisms, the demethylases are divided into two subgroups: the flavin adenine dinucleotide (FAD)-dependent LSD1 and LSD2 and JMJD family containing JmjC domain [3]. Prior to the discovery of the first demethylase LSD1 (also named KDM1A, KIAA0601, BHC110, and AOF2) in 2004 [4], the process of histone methylation is considered to be irreversible. LSD1 specifically demethylates histone lysine residues H3K4me1/2 and H3K9 me1/2 (Fig. 1a). LSD2 (also known as KDM1B or AOF1), a well-known histone H3K4me1/2 demethylase, is the only homolog of LSD1 in human genome and exhibits an overall sequence identity of < 31% with LSD1 [5]. Differently, LSD1 binds at promoter regions, while LSD2 is mainly enriched at the body regions of actively transcribed genes [5]. Therefore, LSD2 is also important in epigenetic regulation but has different structural organization and functions relative to LSD1 [6, 7]. The JMJD family oxidatively removes the trimethyl group of histone lysine residues preferably in a Fe^2+^ and 2-oxoglutarate (2-OG) dependent manner (Fig. 1b) and have key roles in cell differentiation, proliferation, and stem cell self-renewal [8, 9].

LSD1 regulates some non-histone substrates including DNMT1, p53, STAT3, and E2F1 [10], which play vital functions during gene expression [11–15]. These studies indicate LSD1, as an H3K4/9me eraser, could genome-wildly regulated gene expression during carcinogenesis. LSD1 suppresses gene transcription by binding to the CoREST or nucleosome remodeling and deacetylase repressive complex and also promotes transcriptional activation upon binding to androgen receptor (AR) or estrogen receptor (ER) [16], thus regulating numerous fundamental cellular processes [17]. For example, the histone 3 (H3) binding and gene expression of LSD1 is affected by the HDAC1-mediated deacetylation of LSD1. The crosstalk between HDAC1 and LSD1 suggests that the activity of LSD1 may be influenced by HDAC inhibitors [18]. Huang et al. reported that the antitumor activity of HDAC inhibitors against human breast cancer cells was mediated by the crosstalk between LSD1 and histone deacetylases [19].

Elevated levels of LSD1 has been found in diverse cancers and shows close relationship with many cellular effects such as epithelial-mesenchymal transition (EMT), cell proliferation and differentiation, stem cell biology, and malignant transformation [20]. LSD1 inactivation also enhances anti-tumor immunity and inhibits checkpoint [21]. LSD1 dysfunction is also associated with the development of ALL (acute lymphoblastic leukemia) and AML (acute myeloid leukemia) [22–24]. Preclinical studies have revealed that LSD1 inhibition could suppress tumor growth of lung adenocarcinoma independent on driver mutations [25]. Expression profiling reveals that LSD1 inhibition mainly affects replication machinery and cell cycle, and interrupts downstream signaling of EGFR (epidermal growth factor receptor). Pharmacological inhibition of LSD1 leads to inhibition of proliferation, differentiation, invasion, and migration in vitro and in vivo [26]. The combinatory analysis of chromatin immunoprecipitation (ChIP)-Seq and microarray revealed the genes affected by LSD1 inhibition in esophageal squamous cell carcinoma (ESCC) cells [27], in which 17 genes were upregulated and 16 genes were downregulated. In addition to the demethylase activity of LSD1, its demethylase-independent activity is also implicated during carcinogenesis [28–31], this finding may explain the ineffectiveness of catalytic inhibition of LSD1 in some cancers [32, 33]. Targeting the demethylase-independent activity of LSD1 is an emerging strategy for the treatment of cancers. Sehrawat et al. demonstrated that LSD1 promoted AR-independent survival in LSD1 highly expressed castration-resistant prostate cancer (CRPC) cells independent of its demethylase function [31]. Sun and co-authors recently reported that the LSD1/FBXW7 interaction could disrupt FBXW7 dimerization and promote FBXW7 degradation independent of its demethylase activity of LSD1 [29]. Very recently, Vinyard et al. revealed a non-enzymatic role of LSD1 in AML through the CRISPR-suppressor scanning and elucidated that the enzymatic activity of LSD1 was not required for AML survival [28]. Furthermore, LSD1 inhibition can block viral genome transcription and replication of DNA viruses, showing therapeutic potential for the treatment of viral infections [34]. These results highlight the biological importance of LSD1 as an emerging therapeutic target for disease treatment [35]. Currently, numerous natural and synthetic LSD1 inhibitors have been identified in the last decades [36–48], some of which currently undergo clinical assessment for the treatment of AML, SCLC, etc.

## LSD1/KDM1A inhibitors in clinical trials

**Fig. 2:**
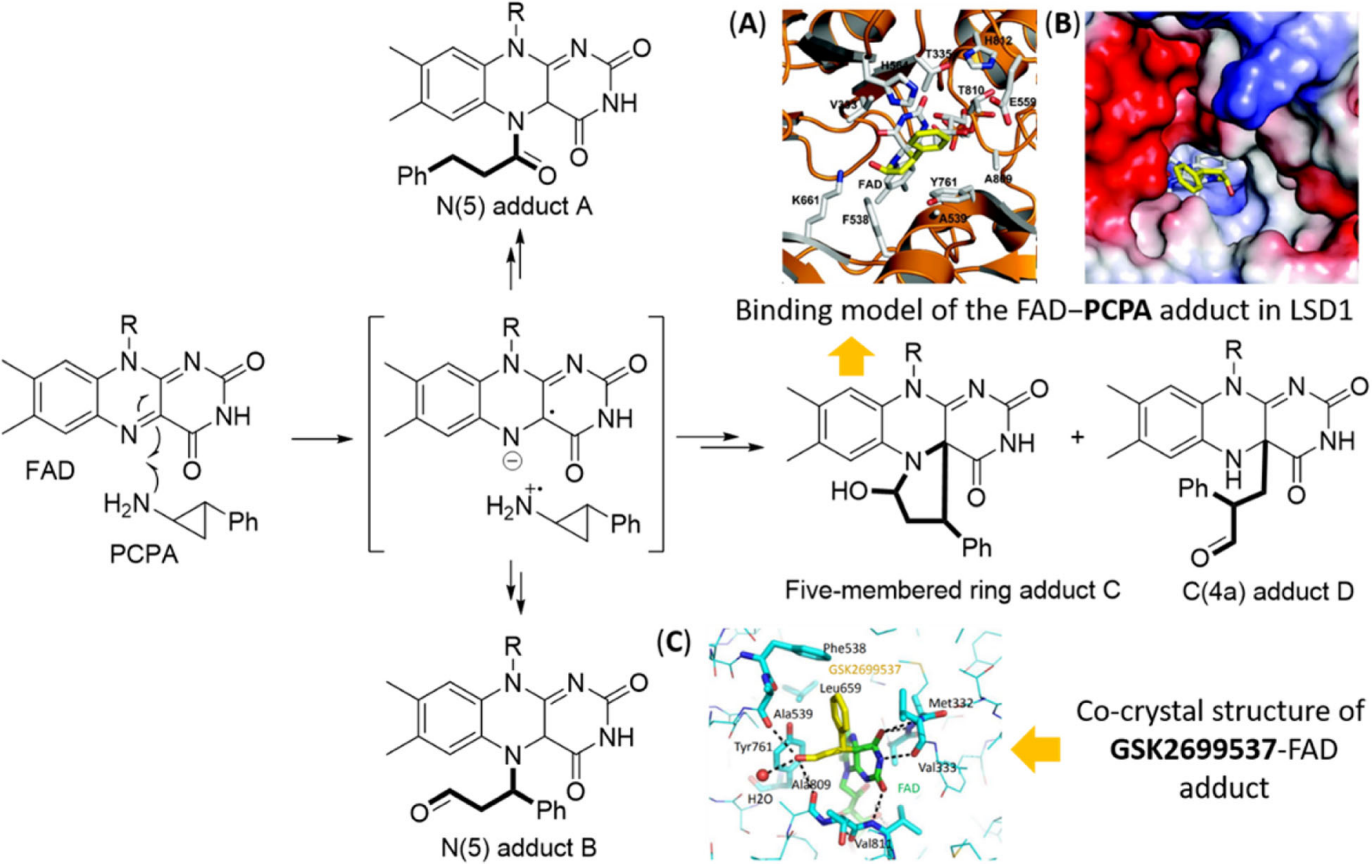
Catalytic mechanisms for LSD1 inhibition with PCPA (fragment derived from PCPA is highlighted in bold). (A) Three dimensional (3D) binding model of the FAD−PCPA adduct with surrounding residues in LSD1 (PDB code: 2UXX); (B) surface map of the FAD−PCPA adduct in LSD1 (PDB code: 2UXX), the positive electrostatic potentials are colored in blue, the negative electrostatic potentials colored in blue red; (C) co-crystal structure of GSK2699537 (gold)-FAD (green) adduct in LSD1/CoREST complex. Figure 2 A–C are excerpted from the references with permissions [50, 52]. Note: The authors claimed they obtained a high-resolution X-ray co-crystal structure of GSK2699537-FAD adduct in their original work [52], but only the related crystallography data were provided in the supporting information, the PDB code is unavailable in RCSB Protein Data Bank (PDB). Recently, a co-crystal structure of human LSD1 in complex with GSK2879552 (PDB code: 6NQU), a structurally close analog of GSK2699537, has been reported [53] and could be for reference

The MAO inhibitor tranylcypromine (TCP) was initially approved by the US Food and Drug Administration (FDA) to treat mood and anxiety disorders in 1961 [49] and subsequently was found to be able to moderately inhibit its homolog LSD1 by forming a covalent adduct with the flavin ring [50, 51]. The identification of TCP as an LSD1 inhibitor has inspired further extensive medicinal chemistry efforts for designing TCP-based irreversible LSD1 inhibitors. Mechanistically, TCP-based LSD1 inhibitors inactivate LSD1 via the single electron reduction mechanism, further homolytic cleavage of the cyclopropyl ring gives different TCP-FAD adducts through distinct pathways (Fig. 2) [50, 52]. As shown in Figs. 2A and B, the phenyl ring of the FAD−PCPA adduct forms weak van der Waals interactions with T335 and T810 but does not form extensive interactions with nearby hydrophobic residues (e.g., Y761, V333, and H564). The structural features suggest that incorporation of hydrophobic substitutions into the phenyl ring would be a viable strategy to design new PCPA-based LSD1 inhibitors with higher potency by forming more interactions with surrounding hydrophobic residues [50]. The covalent modification is also confirmed by the high-resolution co-crystal structure of FAD-GSK2699537 adduct (Fig. 2C) [52].

**Fig. 3:**
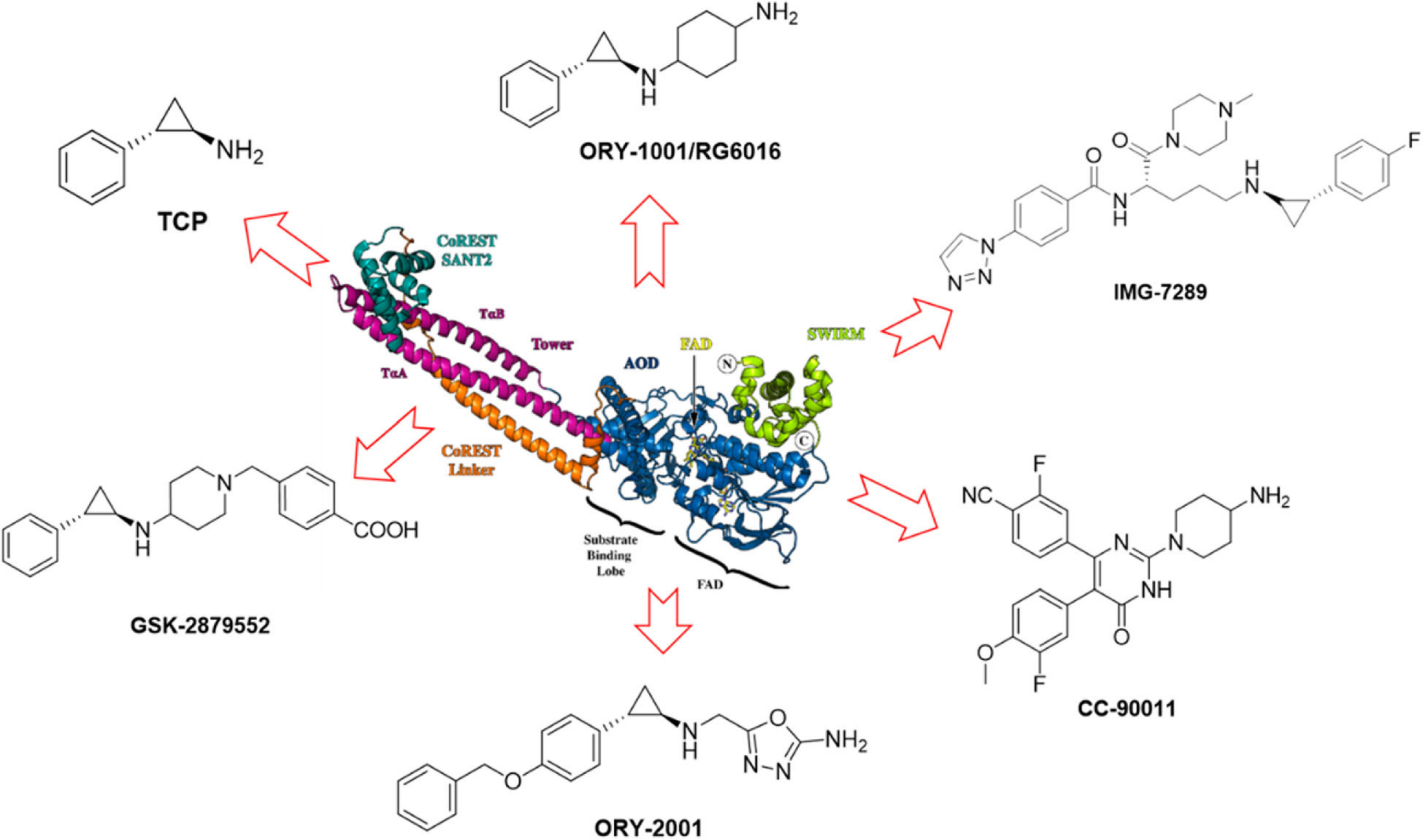
LSD1 inhibitors in clinical trials. The picture showing 3D structure of LSD1 is excerpted from the reference [58]

To date, many irreversible LSD1 inhibitors have been discovered [26, 42], of which TCP, ORY-1001 [54], GSK-2879552 [52, 55], IMG-7289, INCB059872 [56, 57], and ORY-2001 (Vafidemstat) (Fig. 3) presently undergo clinical assessment for cancer therapy. Besides, combined treatment with ATRA (all-*trans* retinoic acid) and Azacitidine are also undergoing clinical investigation for cancer therapy, such as AML, ALL, and SCLC (Table 1). Besides, CC-90011 (Fig. 3), a reversible LSD1 inhibitor, is also being evaluated in clinical trials. Of note, the clinical trials of GSK-2879552 for AML and relapsed/refractory SCLC, respectively, have been terminated because of the risk in relapsed refractory AML and SCLC. Apart from applications in the field of oncology, LSD1 inhibitors ORY-1001, GSK-2879552, IMG-7289, ORY-2001 (dual LSD1/MAO-B inhibitor) also show therapeutic potentials in clinical investigation to treat MDS, myelofibrosis, multiple sclerosis (MS), and Alzheimer’s disease (AD) (Table 1).

**Table 1:** Overview of LSD1/KDM1A inhibitors in clinical trials

Drugs	Phase	Trial number	Diseases	Status
ORY-1001	Phase I/II	NA*	AML	Unknown
	Phase I	NCT02913443	SCLC	Completed
	Preclinical	NA*	AML, solid tumors	Unknown
TCP	Phase I	NCT02273102	AML; MDS	Active, not recruiting
	Phase I/II	NCT02261779	Relapsed/refractory AML	Unknown
	Phase I/II	NCT02717884	Non-M3 AML blasts	Recruiting
GSK2879552	Phase I	NCT02034123	Relapsed/refractory SCLC	Terminated
		NCT02177812	AML	Terminated
	Phase II	NCT02929498	High-risk MDS	Terminated
INCB059872	Phase I/II	NCT02712905	Solid tumors and hematologic malignancy	Recruiting
	Phase I	NCT03514407	Relapsed Ewing sarcoma	Recruiting
	Phase I/II	NCT02959437	Solid tumors Advanced malignancies Metastatic cancer	Active, not recruiting
	Phase I	NCT03132324	Sickle cell disease	Terminated
	Phase I/II	NCT04061421	MDS/MPN	Not yet recruiting
IMG-7289	Phase II	NCT03136185	Myelofibrosis	Recruiting
	Phase II	NCT04081220	Essential thrombocythemia	Not yet recruiting
	Phase I	NCT02842827	AML and MDS	Completed
CC-90011	Phase I	NCT02875223	Relapsed/refractory solid tumors and non-Hodgkin’s lymphomas	Recruiting
	Phase I/II	NCT03850067	SCLC	Recruiting
ORY-2001	Phase I	NA*	Multiple sclerosis	Recruiting
	Phase IIa	NCT03867253	Mild to moderate Alzheimer’s disease	Recruiting

*NA means the related data are not available on the *ClinicalTrials.gov* website and excerpted from the *Oryzon Genomics* website. Updated on October 1, 2019

### TCP (tranylcypromine)

The tranylcypromine (abbreviated as TCP or PCPA), an inhibitor of monoamine oxidase (MAO) used in clinic for the treatment of depression [59, 60], was identified as an irreversible and weak LSD1 inhibitor [51, 61]. Currently, 26 studies have been registered in *clinicaltrials.gov* website under the term “tranylcypromine,” three of them are undergoing for evaluating the therapeutic efficacy against AML and MDS. A phase I/II study was initiated on October 10, 2014, to analyze feasibility, safety, pharmacodynamics, and effectivity of ATRA/TCP treatment in patients with relapsed or refractory AML or in patients with AML who are not eligible for intensive treatment (ClinicalTrials.gov Identifier: NCT02261779). On October 23, 2014, a phase 1 study, sponsored by University of Miami, was also initiated to evaluate the safety and tolerability of TCP/ATRA combination therapy for adult patients with AML and high-grade MDS (ClinicalTrials.gov Identifier: NCT02273102). On March 24, 2016, Michael Luebbert initiated a phase I/II study of sensitization of Non-M3 AML blasts to ATRA by TCP treatment, aiming to determinate the maximum tolerated dose (MTD) of TCP/ATRA and TCP/cytarabine treatment (fixed dose used for ATRA and cytarabine in this study, ClinicalTrials.gov Identifier: NCT02717884).

TCP poorly inhibited LSD1 (K_i_ = 243 μM) by forming covalent TCP-FAD adducts [62]. TCP increased methylation levels of global H3K4, suppressed cell growth of bladder cancer and neuroblastoma, and also showed potency in mouse models [63, 64]. Majello et al. first reported that LSD1, by binding to the promoter region of Sestrin2 (SESN2), regulated autophagy in neuroblastoma (NB) cells, LSD1 inhibition by TCP-induced SESN2 expression that hampered the activity of mTORC1, leading to enhanced autophagy of NB cells [65]. In non-APL AML, TCP unlocked therapeutic response driven by ATRA. LSD1 inhibition increased H3K4me2 and expression of myeloid-differentiation-associated genes, not a genome-wide increase in H3K4me2. In primary human AML cells in vivo in NOD-SCID mice, combined treatment with ATRA and TCP significantly reduced the engraftment [66], suggesting that this combination therapy may target leukemia-initiating cells (LIC). Furthermore, ATRA/TCP combination also had a superior anti-leukemic effect to ATRA or TCP alone in human AML cells in NOD-SCID γ mic. These data strongly suggest that the ATRA/TCP combination therapy may pave a new way for AML.

In the phase 1 study of ATRA/TCP combination (ClinicalTrials.gov Identifier: NCT02273102) [67], all 15 patients received continuous daily dosing of both ATRA (45 mg/m2 in divided doses) and TCP (3 escalating dose levels, 10/20/30 mg BID), with a 3-day lead-in of TCP only during cycle 1 (21 days). The results showed that the combination was well tolerated with an acceptable safety profile in patients with R/R AML and MDS, TCP 20 mg BID was selected as the MTD and the recommended phase 2 dose (RP2D). The most common grade 1/2 treatment emergent adverse effects (TEAEs) were dry mouth (33%), febrile neutropenia (27%), dry skin (27%), fatigue (27%), dizziness (27%), rash (27%), headache (27%), increase in creatinine (27%), and infection (20%), diarrhea (20%), nausea (20%), urinary frequency (20%), vomiting (20%), and thrombocytopenia (20%). Febrile neutropenia (27%) was the most common grade 3/4 TEAE, followed by thrombocytopenia (20%), sepsis (13%), anemia (13%), and lung infection (13%). For the phase 2 study, an intermittent ATRA schedule may be pursued because of the skin toxicity observed in responders receiving continuous exposure to ATRA in current study.

### ORY-1001/iadademstat

ORY-1001 (also named iadademstat, RG6016 and RO7051790) developed by Oryzon Genomics is being investigated in clinical trials for the treatment of AML and solid tumors. The phase 1 clinical trial for relapsed, extensive-stage disease SCLC treatment has been done (ClinicalTrials.gov Identifier: NCT02913443). ORY-1001 potently inactivates LSD1 (IC_50_ < 20 nM) and is highly selective over other FAD-dependent aminoxidases (IL4I1, MAO-A/B, LSD2 > 100 μM, SMOX 7 μM) [68]. ORY-1001 time-/dose-dependently induces accumulation of H3K4me2 at LSD1 target genes and causes concomitant induction of differentiation markers (H3K4me2 and FACS CD11b EC_50_ < 1 nM) in THP-1 (MLL-AF9) cells. ORY-1001 induces cell apoptosis of THP-1 cells, inhibits colony formation and cell proliferation of MV(4;11) (MLL-AF4) cells (EC_50_ < 1 nM) and significantly reduces tumor growth in MV(4;11) xenografts after oral administration of < 0.020 mg/kg daily. ORY-1001 is stable in hepatocytes with the Clint less than 0.6 mL/min/g at 1 μM without inhibition of CYP (IC_50_ > 100 μM) and hERG (< 2% inhibitory rate at 10 μM) and shows excellent oral bioavailability, activity, and target exposure in vivo. A multicenter, first-in-human phase 1 study for evaluating the safety, pharmacodynamics (PD), and pharmacokinetics (PK) studies of ORY-1001 in acute leukemia (EUDRACT 2013-002447-29) shows that ORY-1001 at the recommended dose is well tolerated and promotes differentiation of blast cells in 64% of patients [69]. ORY-1001 plasma concentration increased with dose across cohorts. At 140 μg/m2/day (recommended dose) on day 1 (d1), C_max_ is 13.1 ± 7.2 and AUC_(0-24h)_ is 181.7 ± 61.3 pg.hr/mL. On d5, C_max_ is 42.2 ± 27 and AUC_(0-24h)_ is 723.3 ± 341.5 pg.hr/mL. For 27 subjects in the dose escalation phase, the most frequent adverse drug reaction (ADR) is thrombocytopenia (7 events, 5 subjects). At the end of the dose escalation phase, the most frequent ADRs are asthenia (16 events, 12 subjects), febrile neutropenia (15 events, 13 subjects), constipation (12 events, 9 subjects), and peripheral edema (11 events, 8 subjects).

A recent study showed that co-treatment with ORY-1001 and BET protein inhibitor OTX015 showed synergistic lethality against human AML blast progenitor cells (BPCs) [70]. ORY-1001 is synergistic with standard-of-care drugs (e.g., ATRA, cytosine arabinoside, and quizartinib), selective epigenetic and targeted inhibitors (e.g., EPZ5676, SGC-0946, decitabine, azacitidine, SAHA, and ABT-737) in MV(4;11), MOLM13, and MOLT4 cell lines, suppresses growth of an AML xenograft model, and prolongs survival of a mouse patient-derived xenograft (PDX) model of T cell acute leukemia [71, 72]. Additionally, ORY-1001 shows better growth inhibition against a panel of classic SCLC cell lines compared to variant ones with the IC_50_ values ranging from sub-nanomolar to nanomolar [73]. ORY-1001 treatment inhibits xenograft growth of response signature positive cell line NCI-H510A, but is less sensitive to the NCI-H526 xenografts. More recently, Shan et al. revealed that ORY-1001 inhibited growth and induced apoptosis of lung cancer cells through triggering HK2-mediated Warburg effect [74]. Augert et al. reported that ORY-1001 treatment activated the NOTCH signaling and suppressed ASCL1 expression and SCLC tumorigenesis. In a chemo-resistant PDX model, ORY-1001 treatment-induced NOTCH activation and caused complete and durable tumor suppression [75]. Previous studies have shown that growth factor–independent family (GFI1 and GFI1B) is prevalent oncogenes of group 3 and group 4 medulloblastoma (MB) [76]. Wechsler-Reya et al. recently reported that LSD1 played essential roles in GFI1-mediated transformation of MB by binding to GFI1, pharmacological inhibition of LSD1 with ORY-1001 effectively inhibited growth of GFI1-driven tumors, suggesting therapeutic potentials of LSD1 inhibitors in GFI1-driven MB [77]. Maes et al. highlighted therapeutic potential of ORY-1001 and checkpoint inhibitors for the treatment of melanoma [78]. After co-treatment with ORY-1001 and the anti-PD1 antibody for 22 days, significant tumor growth inhibition (TGI) was achieved, 54% higher than that of the anti-PD1 antibody-treated group. However, a recent study by Shipley et al. revealed that catalytic inhibition of LSD1 with ORY-1001 was ineffective in cell viability and invasion of Ewing sarcoma and desmoplastic small round cell tumors (DSRCT) in 2D and/or 3D assays [32]. The findings suggest that catalytic inhibition of the LSD1 demethylase activity is insufficient in Ewing sarcoma or DSRCT. The demethylase-independent activity of LSD1 should be considered for Ewing sarcoma.

### GSK2879552

Two clinical phase 1 trials investigating the safety, pharmacokinetics, pharmacodynamics, and clinical activity of GSK2879552 in patients with relapsed/refractory SCLC (ClinicalTrials.gov Identifier: NCT02034123) and AML (ClinicalTrials.gov Identifier: NCT02177812) have been terminated. Besides, a phase I/II, open-label study evaluating the safety and clinical activity of GSK2879552 alone, or in combination with azacitidine in subjects with MDS, has also been terminated (ClinicalTrials.gov Identifier: NCT02929498). As shown in the website of *clinicaltrials.gov*, the risk benefit does not favor continuation of these three studies.

GSK2879552 was initially identified from a chemical collection containing 2.5 million compounds [52]. Compared to closely related enzymes including LSD2 and MAO-A/B, ion channels, G protein coupling receptors (GPCR), nuclear receptors, transporters, GSK2879552 showed high selectivity to FAD utilizing proteins including LSD1. GSK2879552 treatment led to complete inactivation of LSD1 over time (LSD1 K_I_^app^ = 1.7 ± 0.5 μM, k_inact_ = 0.11 ± 0.01 min^−1^, k_inact_/ K_I_^app^ = 6.47 × 10^−2^ ± 3.07 × 10^−3^ min^−1^ μM^−1^). GSK2879552 did not modify the protein backbone of LSD1, loss of the characteristic UV absorbance of LSD1-bound FAD suggested covalent modification of LSD1, which was further confirmed by the co-crystal structure of the GSK2879552-FAD adduct (PDB code: 6NQU). In contrast, the free FAD was unaffected, indicating that the demethylation of LSD1 was an enzyme-mediated process. Taken together, the results demonstrate that GSK2879552 is a mechanism-based irreversible LSD1 inhibitor depending on the catalytic activity of the enzyme.

The antitumor screening of GSK2879552 against a panel of cell lines showed that the antitumor activity is mainly restricted to SCLC and AML. GSK2879552 treatment causes local changes near transcriptional start sites of genes whose expression increases with LSD1 inhibition without effects on the global levels of H3K4me1/2 and increased cell surface expression of CD11b and CD86 in AML cell lines. GSK2879552 treatment shows potent anti-proliferative effects in some AML cell lines and also inhibits colony formation of AML blast in primary AML patient-derived marrow samples [52, 79, 80]. Smitheman et al. recently reported that LSD1 inhibitor GSK2879552 is synergistic with ATRA in cell proliferation, markers of differentiation, and cytotoxicity of acute myeloid leukemia across subtypes [81]. Additionally, the SCLC cell lines and primary samples with DNA hypomethylation is sensitive to GSK2879552 treatment, over 80% of tumor growth inhibition (TGI) is observed in mice engrafted with SCLC lines after GSK2879552 treatment [82].

Upon oral administration, GSK2879552 was well tolerated at 1.5 mg/kg in SCLC xenograft bearing mice without loss of body weight or disruption of normal grooming behavior. GSK2879552 showed acceptable PK properties (F% = 59.2%, T_1/2_ = 1.9 h, C_max_ = 720 ng/mL) when 5 mg/kg of GSK2879552 was orally administered [52], allowing for further in vivo studies. Detailed PK data are shown in Table 2. 28-day toxicology studies in rats and dogs showed that GSK2879552 treatment caused severe but reversible toxicities including thrombocytopenia, neutropenia, myelofibrosis, and congestion with and without lymphoid necrosis in lymphoid organs.

**Table 2:** Pharmacokinetics of LSD1 inhibitor GSK2879552 in mice

	C_max_ (ng/mL)	T_max_ (h)	AUC_(0-last)_ (ng*h/mL or ng*h/g)	AUC(0-inf) (hr*ng/mL or hr*ng/g)	DNAUC_(0-last)_ (ng*h/mL/mg/k g)	T_1/2_ (h)	AUC_%Extrap_pred (%)
Blood	720	0.25	852.7	903.2	171	1.9	5.6
Tumor	354.7	0.5	2321.6	2693.0	464	8.4	13.8

### INCB059872

INCB059872 developed by Imago BioSciences currently undergoes four clinical trials for cancer therapy (Table 1). An open-label phase 1b study of the safety, tolerability, and preliminary antitumor activity of INCB059872 is currently under clinical assessment in participants with relapsed or refractory Ewing sarcoma (Trial Identifier: NCT03514407 and EudraCT 2018-000062-11). A phase 1/2, open-label, dose-escalation/dose-expansion, safety, and tolerability study of INCB059872 in subjects with advanced malignancies has been initiated since May 2016 (ClinicalTrials.gov Identifier: NCT02712905). This studies include four parts: (A) to determine the recommended dose(s) of INCB059872; (B) to determine the safety, tolerability, efficacy, PK, and PD of the selected monotherapy dose(s) in different types of tumors such as AML/MDS, SCLC, myelofibrosis, Ewing sarcoma, and poorly differentiated neuroendocrine tumors; (C) to determine the recommended dose(s) of INCB059872 in combination with azacitidine and ATRA in AML and in combination with nivolumab in SCLC; (D) to further determine the safety, tolerability, efficacy, PK, and PD of the selected combination dose(s). The open-label, phase 1/2 study in subjects with advanced or metastatic solid tumors (ClinicalTrials.gov Identifier: NCT02959437) aims to evaluate the safety and tolerability of the combination therapies of INCB059872 with pembrolizumab and epacadostat. Aster Pharmaceuticals, Theradex, and Incyte Corporation plan the phase I/II ABNL-MARRO trial for myelodysplastic syndromes or myeloproliferative disorders in USA in October 2019 (ClinicalTrials.gov Identifier: NCT04061421). However, a phase 1 study evaluating the safety, pharmacokinetic, and biological activity of INCB059872 in subjects with sickle cell disease has been terminated on March 1, 2019, due to a business decision not to pursue INCB059782 in sickle cell disease indication (ClinicalTrials.gov Identifier: NCT03132324).

In AACR Annual Meeting 2016, Lee et al. reported that INCB059872, a new FAD-directed LSD1 inhibitor, inactivated LSD1 by forming covalent FAD-adducts, potently and selectively inhibited cell proliferation against SCLC cells (EC_50_: 47~377 nM) [57]. In contrast, non-tumorigenic IL-2 stimulated T cells from normal donors were less sensitive to INCB059872 (IC_50_ > 10 μM). Oral administration of INCB059872 inhibited tumor growth of SCLC xenograft models bearing NCI-H526 and NCI-H1417, induced FEZ1 and UMODL1 genes in these models, and significantly reduced serum levels of the neuroendocrine marker pro-GRP in the NCI-H1417 human SCLC xenograft model. Preclinical studies evaluating the antitumor efficacy of combined treatment of INCB059872 with standard of care therapies for SCLC are also under evaluation. Meanwhile, Lee et al. also reported the antitumor efficacy of INCB059872 in preclinical models of human and murine AML [56]. INCB059872 induced growth inhibition and differentiation, induction of myeloid differentiation markers CD86 and CD11b was observed in various human AML cell lines and also in human AML xenograft models (confirmed by PD studies). INCB059872 significantly inhibited tumor growth of human AML xenograft models and prolonged the median survival of MLL-AF9 expressing leukemic mice. Mechanistic studies demonstrated that INCB059872 induced cell differentiation of murine blast cells, reduced blast colonies, and normalized clinical hematological parameters to those of non-leukemic mice. Notably, in both murine MLL-AF9 leukemic model and the human AML xenografts, INCB059872 achieved maximal antitumor efficacy with both dosing regimens of daily (QD) and alternative-day (QoD). In AACR Annual Meeting 2018, Chadderton et al. further reported their findings that INCB059872 could increase myeloid differentiation in human AML PDX (patient-derived xenografts) models and primary AML samples, accompanied by increasing populations of CD14+ and CD15+ [83]. Furthermore, INCB059872 induced the differentiation of CD34+/CD38- to CD34+/CD38+, which in turn gave rise to lineage-specific progenitors in the human AML PDX models. Both studies support that INCB059872 is a promising epigenetic agent for AML therapy.

LSD1 controls the fate of pluripotent cancer stem-like cells (CSCs), its amplification has been associated to tumorigenic and CSC-like features [84, 85]. In AACR Annual Meeting 2018, Civenni et al. reported the antitumor efficacy of INCB059872 against prostate CSCs, mainly focusing on its effects on the growth properties, self-renewal, and tumorigenic capability [86]. In ex vivo tumor-sphere assays, INCB059872 strongly inhibited growth of tumor-initiating stem-like cells isolated from prostatic tumors and also suppressed formation of tumor sphere and colony by human prostate cancer cells. However, the effects of INCB059872 on cell proliferation and viability of bulk tumor cells were limited, long-term exposure was required for effectiveness. These effects were also observed in several prostate cancer cells, independent on the status of androgen receptor (AR) and genetic features. Importantly, LSD1 knockdown had similar effects of INCB059872. These data confirmed the essential role of LSD1 in maintaining the stem-like and tumorigenic subpopulation of prostate tumors, and pharmacological inhibition of LSD1 by INCB059872 could reduce self-renewal and survival capability of prostate CSCs. The results suggest that LSD1 inactivation by INCB059872 could offer a new strategy for treating prostate cancer.

### IMG-7289/Bomedemstat

IMG-7289, an investigational small-molecule therapeutic agent developed by Imago BioSciences, is currently undergoing phase 1 clinical assessment at multiple locations in Australia for myelofibrosis (MF) treatment (ClinicalTrials.gov Identifier: NCT03136185). IMG-7289 alone or combined treatment with ATRA has also entered phase IIa clinical trial for treating high-risk AML and MDS (ClinicalTrials.gov Identifier: NCT02842827). In 2018, the IND application of IMG-7289 has been accepted by FDA to carry out the clinical development for myelofibrosis (please refer to IMG-7289 at AdisInsight website, https://adisinsight.springer.com/drugs/800048131). Very recently, a single-center, open-label phase 2 clinical trial (ClinicalTrials.gov Identifier: NCT04081220) sponsored by the University of Texas Health Science Center at San Antonio was initiated, aiming to evaluate the effects of IMG-7289 administered orally once daily in patients with essential thrombocythemia (ET).

IMG-7289 irreversibly inhibits LSD1, increases H3K4 and H3K9 methylation, and then alters gene expression (for details, please refer to LSD1 inhibitor IMG-7289 at National Cancer Institute, NIH). IMG-7289 inhibits the production of inflammatory cytokines, impairs self-renewal and proliferation of neoplastic stem cells, and shows significant disease-modifying activities in multiple non-clinical models of myelofibrosis. In non-clinical models, LSD1 inhibition could suppress self-renewal of neoplastic stem cells such as those in AML and MF. Across a range of myeloid malignancy models, IMG-7289 alone or in combination with other anti-neoplastic agents, demonstrated robust strong in vivo activity (please see IMG-7289 at Imago BioSciences website for details). Jonas Samuel Jutzi et al. reported that IMG-7289 treated mice showed drastic decreases in platelet count, reticulocytes, monocytes and neutrophils as well as increased global H3K9me2 levels in the bone marrow compared to control mice. IMG-7289 normalized or stabilized elevated complete blood counts (CBCs) in a JAK2^*V617F*^ mouse model of myeloproliferative neoplasms (MPNs), decreased JAK2 mutant allele burden, pro-inflammatory cytokine levels, and conferred a clear survival advantage [87]. The combination of IMG-7289 with JAK1/2 inhibitors might accelerate treatment effects. The PD/PK and adverse side reactions have not been released currently.

### ORY-2001/Vafidemstat

ORY-2001 (Vafidemstat), a dual LSD1/MAO-B inhibitor developed by Oryzon Genomics, has recently been approved to enter IIa clinical trial to evaluate the safety, tolerability, and preliminary efficacy of ORY-2001 in patients with mild to moderate Alzheimer’s disease (ClinicalTrials.gov Identifier: NCT03867253). ORY-2001 is also clinically tested at IIAa Phase for the treatment of RRMS (relapsing-remitting multiple sclerosis) or SPMS (secondary progressive multiple sclerosis) (https://www.oryzon.com/sites/default/files/PRESS_RELEASE_03-2018.pdf). It is the first epigenetic approach in multiple sclerosis (MS), representing a new avenue for clinical development of ORY-2001 in different neurological indications.

ORY-2001 is an orally active and blood-brain barrier (BBB)–permeable therapeutic agent that shows excellent selectivity to LSD1 and its homology MAO-B over chromatin modulators, other amine oxidases containing FAD, and 100 targets from the CEREP diversity panel. For Huntington’s disease, ORY-2001 is effective in preventing development of cognitive impairment in the R6/1 model and the SAMP-8 mice [88]. ORY-2001 restores behavioral deficits and UCHL1 (ubiquitin carboxyl-terminal esterase L1) and Notch1 levels in SAMP8 mice, a model for aging and AD [89]. Buesa and colleagues reported that ORY-2001 treatment could downregulate S100A9, which is overexpressed in patients with postoperative cognitive dysfunction, AD, and traumatic brain injury. Compared to the MAO-B inhibitor rasagiline (3 mg/kg), ORY-2001 could significantly prevent the progression of memory deficit completely in SAMP-8 mice at doses ranging from 0.3 to 3 mg/kg [90]. Besides, ORY-2001 was more effective than fingolimod in reducing the clinical score in the EAE (experimental autoimmune encephalomyelitis) mouse model. Both compounds induced IL2, increased IL-4, IL-10, IP-10, and MCP1, and enhanced cellularity in lymph nodes. In contrast, ORY-2001 increased cellularity in the spleen and modulated compartment of B cells. Gene expression profiling showed that ORY-2001 reduced S100a9 expression in the spinal cord, induced *transthyretin*, and reduced the demyelination marker cystatin F [91].

In the first-in-human clinical trial of healthy individuals (over 100) and AD patients, ORY-2001 was well tolerated and no clinically significant changes, physical findings, ECGs (electrocardiogram), and vital signs were observed up to 4 mg in SAD (a single ascending dose) and up to 2.5 mg in MAD (multiple ascending dose) subgroups [92]. Of particular interest was the hematological safety of ORY-2001, no hematological side effects were observed in the SAD subgroup; reversible platelet reduction in the MAD subgroup was observed at the dose of 2.5 mg in two out of eight patients. However, ORY-2001 treatment at 4 mg caused transient thrombocytopenia, headache episodes, and a platelet rebound. ORY-2001 exhibited good PK/PD profiles with fast oral absorption, a long T_1/2_ (22 h), moderate systemic accumulation (mean AUC ratio < 2) after 5 days of administration. In PD tests, ORY-2001 showed the dose-dependent target engagement (T_1/2_~84 h) in peripheral blood mononuclear cells (PBMCs).

### CC-90011

CC-90011 developed by Celgene is the first reversible LSD1 inhibitor in clinical trials and has proven to be effective in advanced solid tumors and R/R NHL (relapsed/refractory non-Hodgkin’s lymphoma), particularly in patients with neuroendocrine tumors (NETs) [93, 94]. CC-90011 currently undergoes phase 1 clinical trial for safety and efficacy evaluation in patients with relapsed/refractory solid tumors and NHLs (non-Hodgkin’s lymphomas) (Clinical trial identification: NCT02875223 and EUDRACT 2015-005243-13). Recently, the Celgene initiated a phase 1/2 studies to evaluate the safety, tolerability, and preliminary efficacy of combined treatment of CC-90011 with cisplatin or etoposide in patients with first line, extensive stage small cell lung cancer (ClinicalTrials.gov Identifier: NCT03850067). CC-90011 has dose-proportional pharmacokinetics and no dose-limiting toxicities are reported. In phase I study of CC-90011 [93, 94], 50 patients (pts) were enrolled. Forty percent of patients suffered from serious adverse events (AEs) and 6% were treatment-related; the most common grade 3/4 treatment-related AEs were thrombocytopenia (16%) and neutropenia (8%). Peak plasma concentrations were 2–4 h post-dose and average terminal half-life was approximately 60 h. Preliminary pharmacodynamics (PD) studies showed decreased chromogranin A (CgA) levels and MTD in response to CC-90011, correlating with clinical benefit. Blood biomarker analyses showed that higher CC-90011 doses, to a larger extent, suppressed expression of MMD and MYL9, suggesting target engagement to LSD1 at doses ≥ 40 mg. The structure of CC-90011 also complies with our previously proposed “2 + 1” model for LSD1 inhibitor design [35, 95].

## Conclusions and outlooks

In 2004, LSD1 was first identified by Prof. Yang Shi and subsequently found to have important biological roles in diverse biological processes and diseases including cancers and virus infections. Elevated levels of LSD1 have been found during carcinogenesis, in AML and SCLC. Pharmacological inhibition of LSD1 with small molecules has proven to suppress cancer cell differentiation, proliferation, invasion, migration, *etc.* Therefore, LSD1 is becoming an emerging therapeutical target for anticancer treatment [96]. In light of its biological importance of LSD1, numerous LSD1 inhibitors have been reported, including natural products, peptides, and synthetic compounds. TCP has been recognized as a privileged scaffold for designing new irreversible LSD1 inhibitors [26, 42]. To date, some TCP-based irreversible LSD1 inhibitors alone or combination therapy with other therapeutic agents (Table 1) are presently being investigated in clinical trials for disease treatment. As shown in Fig. 3 and associated with our previous review focusing on the TCP analogs as LSD1 inhibitors [26], we can see that relative to TCP, other LSD1 inhibitors including ORY-1001, GSK2879552, IMG-7289, and ORY-2001 exhibit significantly improved potency and selectivity, suggesting that modifications on the phenyl ring and the amine group of the TCP scaffold are crucial for the potency. Here, we also would like to highlight the importance of the TCP scaffold for the anti-LSD1 activity, which forms covalent adduct with FAD (Fig. 2). An additional amine group linked to the NH_2_ group of TCP is beneficial for the activity by forming electrostatic interactions with the negatively charged residues at the entrance of the substrate cleft [97]. Please refer to our previous review for comprehensive structure-activity relationship (SAR) analysis on TCP-based LSD1 inhibitors [26].

Generally, covalent inhibitors have long-lasting effects on the target of interest but may also have promiscuous effects for non-specific irreversible inhibitors, thus leading to adverse drug reactions [16]. As stated in the “LSD1/KDM1A inhibitors in clinical trials” section of this manuscript, the toxicities or side effects of LSD1 inhibitors in clinical trials have been observed in patients. To achieve appreciated clinical outcomes of LSD1 inhibitors, emphasis should be placed at least on the following aspects: (A) Design of appropriate dosing regimens in clinical trials; (B) in-depth mechanistic studies in vitro and in vivo of LSD1 inhibitors, which in turn provide guidance on the design of dosing schedules; (C) development of highly potent and selective reversible LSD1 inhibitors, which may offer safer profiles. Notably, CC-90011 is the only reversible LSD1 inhibitor in clinical trials for cancer therapy, shedding light on the therapeutic potential of reversible LSD1 inhibitors. In structure, CC-90011 complies with our previously proposed “2 + 1” model for designing LSD1 inhibitors [35, 95]. This model could be used for designing new reversible LSD1 inhibitors. Apart from LSD1 inhibitors depicted in Fig. 3, some other LSD1 inhibitors also show promise for cancer therapy. For example, SP-2509, identified by Sharma et al. through the high-throughput virtual screening, is a highly potent, reversible, and non-competitive LSD1 inhibitor (K_i_ = 31 nM, IC_50_ = 13 nM), shows high selectivity over MAO-A/B (IC_50_ > 300 μM), and strongly inhibits proliferation against a panel of cancer cell lines [98]. Subsequently, Sonnemann et al. reported that the cellular response of SP-2509 in AML cells was dominated by the off-target effects of SP-2509 [33]. Sehrawat et al. demonstrated that SP-2509 acted as an allosteric LSD1 inhibitor by targeting a H3 pocket within LSD1 and suppressed tumor growth in castration-resistant prostate cancer (CRPC) preclinical models independent of its demethylase function [31]. Organometallic complexes possess great structural diversity of geometrical shapes and thus have emerged as promising scaffolds for antitumor leads [99]. Yang et al. reported the first rhodium (III)-based LSD1 inhibitor (IC_50_ = 40 nM, K_i_ = 0.57 μM), which showed selectivity over other related enzymes, including KDM2b, KDM7, and MAO. In human prostate cancer cells, this metal complex disrupted the LSD1-H3K4me2 interaction and enhanced the amplification of p21, FOXA2, and BMP2 gene promoters [100]. Natural products are rich sources for identifying bioactive compounds, some natural products have been found to be able to inhibit histone demethylases [39, 101–104]. Representative examples are polymyxins B and E, which inhibit LSD1-CoREST (Ki ~ 157 to 193 nM) by binding to the negatively charged residues at the entrance of the H3 tail-binding cleft [97]. Other privileged scaffolds with promising anti-LSD1 activity include 3-(piperidin-4-ylmethoxy)pyridine [105], thieno[3,2-*b*]pyrrole-5-carboxamide [106, 107], aryl thiourea [41, 43, 108], triazole-fused pyridine [38, 45–47], and dithiocarbamate [40, 109], xanthine [110, 111], etc. Clearly, more reversible LSD1 inhibitors will be developed to evaluate their therapeutic potential in the near future.

LSD1 is involved in many signaling pathways and acts together with other proteins. Therefore, combined treatment of LSD1 inhibitors and other therapeutic targets or dual inhibition of LSD1 and other disease-related proteins may have synergistic effects. Currently, combination therapy of TCP/ATRA, TCP/cytarabine, INCB059872/azacitidine, INCB059872/ATRA, INCB059872/pembrolizumab, INCB059872/epacadostat, IMG-7289/ATRA, CC-90011/cisplatin, and CC-90011/etoposide are under investigation for cancer therapy (Table 1). Besides, the dual LSD1/MAO-B inhibitor ORY-2001 is also under assessment for the treatment of AD, RRMS, and SPMS. These ongoing clinical studies may provide a new direction for epigenetic treatment. Evidently, deep understandings of molecular mechanisms will definitely help us design more appropriate combined therapy. LSD1 forms a complex with HDAC1/2 and CoREST, which stimulates the activity of LSD1 toward nucleosomes. Fiskus et al. reported that co-treatment with SP-2509 and the pan-HDAC inhibitor (HDI) panobinostat (PS) significantly inhibited viability of primary AML BPCs and improved survival of NOD-SCID-γIL-2 receptor-deficient (NSG) mice with established human AML [112]. Furthermore, combined treatment with PS and SP-2509 significantly improved the survival of the mice engrafted with the human AML cells, no any toxicity was observed for this combined therapy [113]. These findings show promise on the combination therapy of LSD1 inhibitor and pan-HDI for AML. Recently, some novel LSD1-HDAC dual inhibitors have been reported, these dual inhibitors, relative to LSD1 or HDAC inhibitor alone, could have superior clinical outcomes and offer unique therapeutic opportunities for cancer treatment [114–117]. Ishikawa et al. reported that the LSD1 inhibitor T-3775440 and the NEDD8-activating enzyme (NAE) inhibitor pevonedistat had synergistic anti-AML effects via transdifferentiation and DNA re-replication in vitro and in vivo, suggesting that dual inhibition of LSD1/NAE represents a novel therapeutic strategy for AML [24]. Domatinostat (4SC-202), a class I histone deacetylase/LSD1 dual inhibitor, is currently under assessment in phase I trial in patients with advanced hematological malignancies [118]. Wobser et al. recently reported that 4SC-202 directly inhibited microtubule formation and effectively suppressed growth of cutaneous T cell lymphoma (CTCL) cells [119]. Studies have showed that LSD1 mediates epidermal growth factor (EGF) signaling, LSD1 knockdown or inhibition suppressed both intrinsic and EGF-induced cell migration in SKOV3 and HO8910 cells [120]. Very recently, we first reported that osimertinib (AZD9291), a third-generation EGFR inhibitor used in clinic, was able to inhibit LSD1 (IC_50_ = 3.98 μM) and showed anti-LSD1 activity at cellular levels [48]. These findings suggest that osimertinib could serve as a hit compound for designing LSD1 and EGFR dual inhibitors for anti-NSCLC drug discovery.

Apart from the demethylase activity of LSD1, its demethylase-independent activity has been found to play important roles during carcinogenesis [28–31], this finding may be responsible for insufficiency of catalytic inhibition of LSD1 in some cancers [32, 33]. Development of small molecules regulating the demethylase-independent activity of LSD1 may provide novel approaches for cancer therapy.

## Data Availability

Not applicable.

## Abbreviations

LSD1: Lysine specific histone demethylase 1
EGFR: Epidermal growth factor receptor
TCP: Tranylcypromine
SCLC: Small lung cancer cells
AML: Acute myeloid leukemia
FAD: Flavin adenine dinucleotide
JMJD: Jumonji C domain-containing
DNMT1: DNA-methyltransferase 1
STAT3: Signal transducer and activator of transcription 3
E2F1: E2F transcription factor 1
2-OG: 2-Oxoglutarate
ALL: Acute lymphoblastic leukemia
FDA: US Food and Drug Administration
PCPA: Trans-2-phenylcyclopropylamine
MAO: Monoamine oxidase
ATRA: All trans retinoic acid
LIC: Leukemia-initiating cells
CYP: Cytochrome P450
hERG: Human ether-a-go-go related gene
PD: Pharmacodynamics
PK: Pharmacokinetics
BET: Bromodomain and extraterminal domain
BPCs: Blast progenitor cells
PDX: Patient-derived xenograft
MB: Medulloblastoma
TGI: Tumor growth inhibition
DSRCT: Desmoplastic small round cell tumors
GSK: GlaxoSmithKline
GPCR: G protein coupling receptors
MF: Myelofibrosis
CBCs: Complete blood counts
MPNs: Myeloproliferative neoplasms
RRMS: Relapsing-remitting multiple sclerosis
SPMS: Secondary progressive multiple sclerosis
MS: Multiple sclerosis
BBB: Blood-brain barrier
UCHL1: Ubiquitin carboxyl-terminal esterase L1
EAE: Experimental autoimmune encephalomyelitis
SAD: Single ascending dose
MAD: Multiple ascending dose
R/R NHL: Relapsed/refractory non-Hodgkin’s lymphoma)
NETs: Neuroendocrine tumors
NHLs: Non-Hodgkin’s lymphomas
